# Mycetoma of the Foot—Diagnosis of the Etiologic Agent and Surgical Treatment

**DOI:** 10.4269/ajtmh.14-0651

**Published:** 2015-07-08

**Authors:** Tiago Mestre, Raquel Vieira, José Coutinho

**Affiliations:** Department of Dermatology and Venereology, Hospital Curry Cabral—Centro Hospitalar Lisboa Central, Lisbon, Portugal; Department of General Surgery, Hospital Curry Cabral—Centro Hospitalar Lisboa Central, Lisbon, Portugal

**Figure 1.**
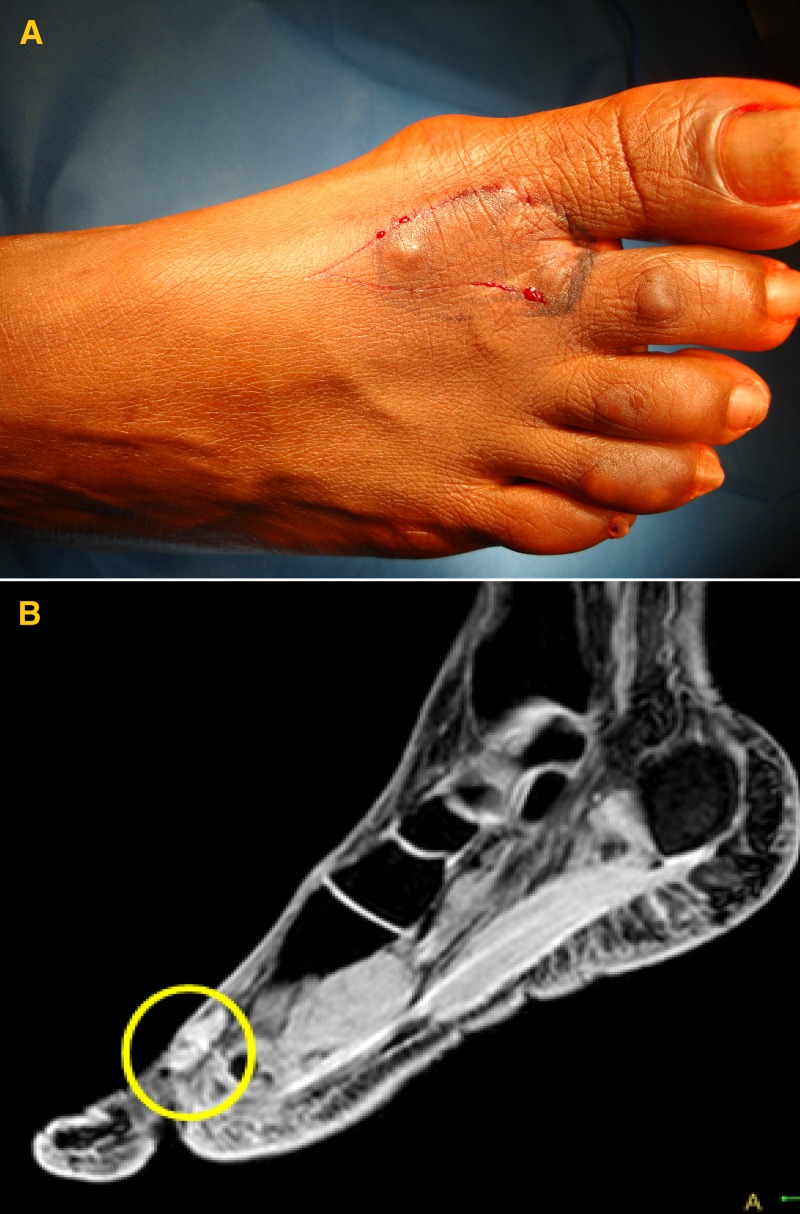
(**A**) Nodular lesion located on the right foot. (**B**) Magnetic resonance imaging (MRI) showed two elongated nodules in subcutaneous fat without bone or tendon involvement.

A 43-year-old female born in Cape Verde presented with a nodular lesion of 2 cm on her right foot. She was admitted because of a recurrence within 1 year after surgery. The magnetic resonance imaging (MRI) showed two elongated nodules in subcutaneous fat (Figure 1). Excision with wide margins was planned and the surgical defect was corrected by a skin graft.

**Figure 2.**
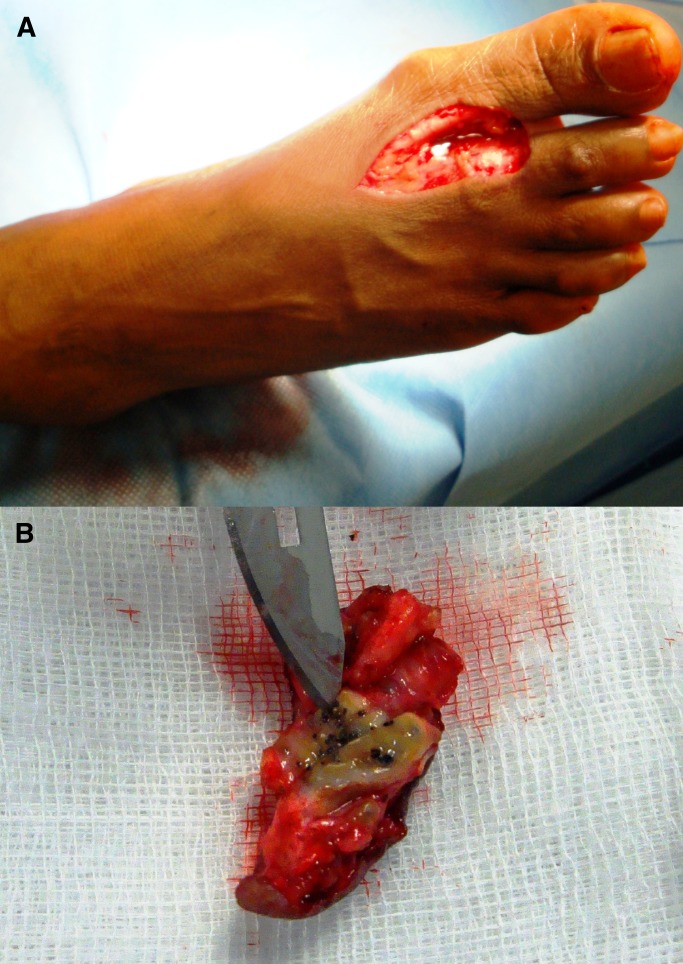
(**A**) Excision with wide margins and the surgical defect was corrected by a skin graft. (**B**) Macroscopically, we could observe black grains with hard consistency measuring 1–2 mm.

**Figure 3.**
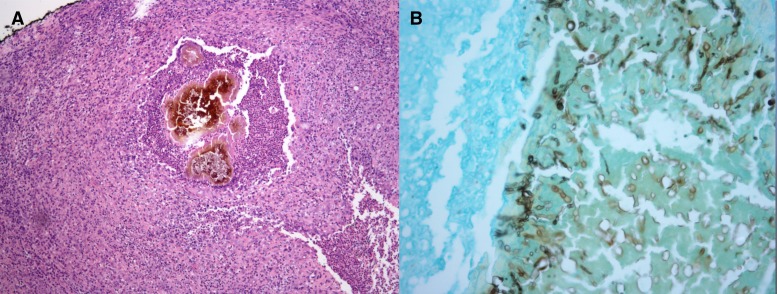
(**A**) (H&E 100×), (**B**) (May-Grünwald-Giemsa 400×) The histopathologic examination showed a chronic granulomatous inflammation with fibrosis and rounded or trilobed grains composed of compact filaments with chlamydospores.

Macroscopically, we observed black grains with hard consistency measuring 1–2 mm (Figure 2). The fungi grew in Brain Heart Infusion Agar and Sabouraud Agar, with the best growth at 37°C. Colonies were grayish brown, folded leathery with dark brown pigment diffusing in the culture medium. The histopathologic examination showed a chronic granulomatous inflammation with fibrosis and rounded or trilobed grains composed of compact filaments with chlamydospores (Figure 3).

Molecular diagnosis was made by the analyses of the internal transcribed spacer region of ribosomal gene sequences. A posterior Basic Local Alignment Search Tool (BLAST) analysis and alignment with reference sequences at GenBank–National Center for Biotechnology Information (NCBI) allowed us to identify the fungi as *Madurella mycetomatis*. After surgery, the patient was treated with itraconazole 400 mg/day for 16 months without any signs of recurrence or drug toxicity.

Eumycetomas are rare in Europe. A multidisciplinary team and long follow-up is needed to ensure the effectiveness of treatment.^1^
